# Emergency Medicine Residency Boot Camp Curriculum: A Pilot Study

**DOI:** 10.5811/westjem.2015.1.23931

**Published:** 2015-03-17

**Authors:** Ramsey Ataya, Rahul Dasgupta, Rachel Blanda, Yasmin Moftakhar, Patrick G. Hughes, Rami Ahmed

**Affiliations:** * Northeast Ohio Medical University, Rootstown, Ohio; † Georgetown University, Washington, District of Columbia; ‡ Summa Akron City Hospital, Akron, Ohio

## Abstract

**Introduction:**

Establishing a boot camp curriculum is pertinent for emergency medicine (EM) residents in order to develop proficiency in a large scope of procedures and leadership skills. In this article, we describe our program’s EM boot camp curriculum as well as measure the confidence levels of resident physicians through a pre- and post-boot camp survey.

**Methods:**

We designed a one-month boot camp curriculum with the intention of improving the confidence, procedural performance, leadership, communication and resource management of EM interns. Our curriculum consisted of 12 hours of initial training and culminated in a two-day boot camp. The initial day consisted of clinical skill training and the second day included code drill scenarios followed by interprofessional debriefing.

**Results:**

Twelve EM interns entered residency with an overall confidence score of 3.2 (1–5 scale) across all surveyed skills. Interns reported the highest pre-survey confidence scores in suturing (4.3) and genitourinary exams (3.9). The lowest pre-survey confidence score was in thoracostomy (2.4). Following the capstone experience, overall confidence scores increased to 4.0. Confidence increased the most in defibrillation and thoracostomy. Additionally, all interns reported post-survey confidence scores of at least 3.0 in all skills, representing an internal anchor of “moderately confident/need guidance at times to perform procedure.”

**Conclusion:**

At the completion of the boot camp curriculum, EM interns had improvement in self-reported confidence across all surveyed skills and procedures. The described EM boot camp curriculum was effective, feasible and provided a foundation to our trainees during their first month of residency.

## INTRODUCTION

As medical students graduate and enroll into their respective residency programs, many realize there is a disconnect between their academic knowledge of medicine and its clinical application in team-based settings.^1^ This disconnect also creates a steep learning curve for interns performing various procedural skills. With the advent and increasing prevalence of simulation training across healthcare institutions, first- year residents are able to better assimilate into their programs and treat patients with a consistent standard of care, decreasing the “July Effect.”^2^ Healthcare systems, recognizing the integral role of simulation training, have begun organizing “boot camps,” curricula that involve a series of training sessions and debriefings with the intent of not only increasing the confidence of resident physicians, but also standardizing a level of competency and performance across various procedures. Even though the integral role of boot camps has been understood in resident training, barriers exist nationally when it comes to implementing them. Boot camps are still a transitioning aspect of medical training.^3^ Great ambiguity exists in defining and implementing boot camp curricula.

Emergency medicine (EM) residents, who are responsible for triaging, diagnosing and stabilizing patients, must be proficient in a large scope of procedures, ranging from airway management to lumbar puncture. They must also develop the ability to lead interprofessional teams during high acuity resuscitations. Therefore, establishing a boot camp curriculum for these residents is of paramount importance.

In this article, we describe the development of a broad-based EM boot camp curriculum. This includes the various materials and methodology used and measures the confidence of interns through a pre and post survey.^4^ Ultimately, we aim to provide a framework through which other EM residencies can implement boot camp programs of their own.

## METHODS

### Study and Boot Camp Curriculum Design

**Figure 1:**
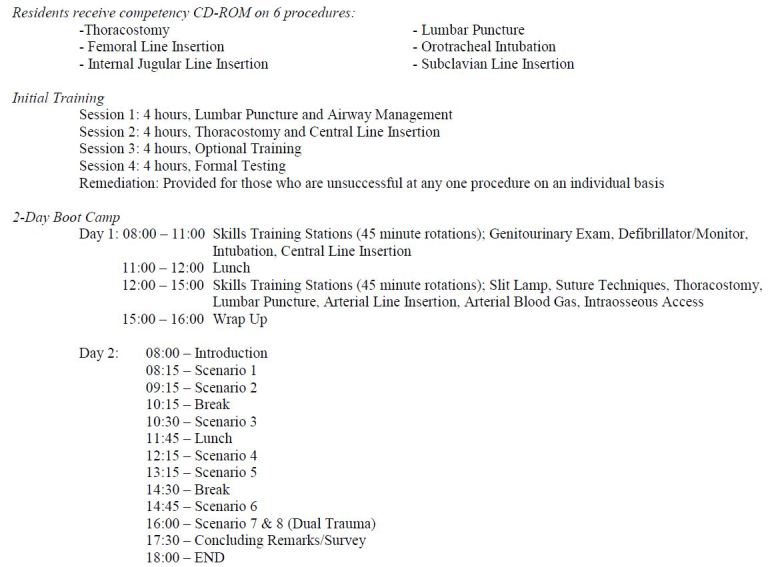
Boot camp curriculum overview.

We designed a one-month boot camp curriculum with the intention of improving the confidence, procedural performance, leadership, communication and resource management of EM interns. Our comprehensive curriculum consisted of approximately 12 hours of initial training and culminated with a two-day, 16-hour capstone experience (Figure 1). Initial training required approximately eight hours for set up and breakdown of equipment, printing and grading of checklists, and remediation testing. Prior to executing the capstone boot camp experience, 20 to 24 hours of initial preparation from seasoned simulation faculty was necessary for curriculum development including goals and objectives, simulation case development, and retrieval of multimedia images. After initial preparation was completed, a walk through and rehearsal was performed by simulation faculty and key staff to ensure cases ran as planned and necessary equipment was available for each scenario (four to six hours). In total, approximately 32–38 hours of preparation were required to execute this curriculum.

### Initial Training

During orientation and prior to their initial simulation training, each intern received a CD-ROM containing reading materials, videos and internally developed competency checklists on six procedures (Figure 1). Interns were required to complete the appropriate readings and score a minimum of 80% on an online multiple choice test for each procedure as well as a formal summative evaluation to demonstrate procedural competence on the simulator. Residents had to correctly perform the procedure with a minimum of 80% of all competency checklist items including all items designated as critical actions. Initial training included two four-hour sessions of hands-on instruction, four hours of optional training, and four hours of formal testing (Figure 1).

### Two-Day Capstone Experience

#### Day 1 - 8 hours of instruction

**Figure 2:**
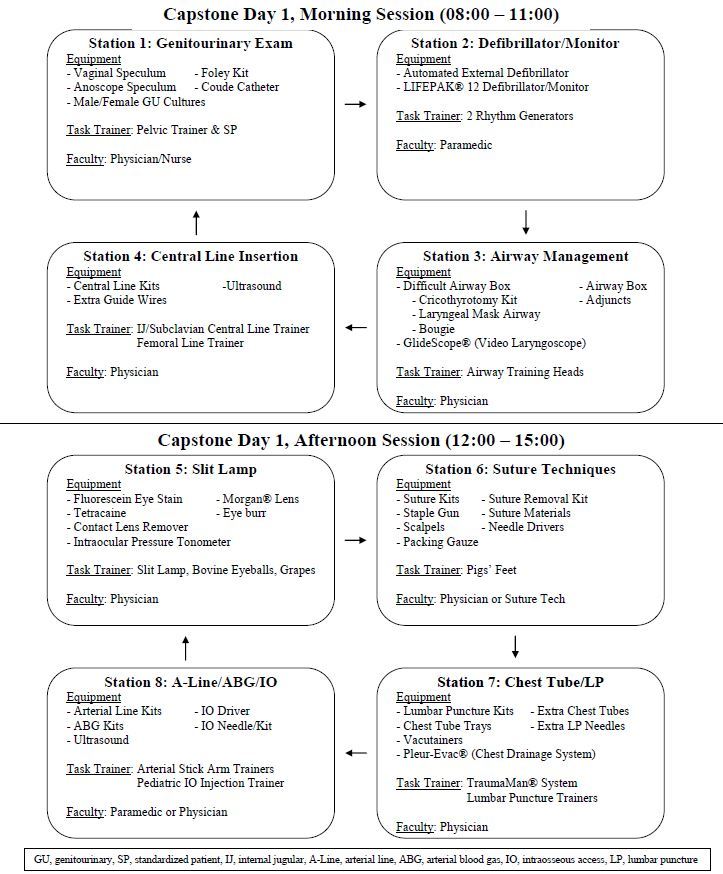
SKill Stations and required equipment for capstone experience day 1. *GU,* genitourinary, *SP*, standardized patient; *IJ*, internal jugular, *A-line,* arterial line; *ABG*, arterial blood gas; *IO*, intraosseous access; *LP,* lumbar puncture

Two weeks after initial training, twelve EM interns participated in the two-day capstone experience. During the first day, four groups of three residents rotated through eight skills stations at 45- minute intervals (Figure 2). At each station, a physician or a simulation team member demonstrated the designated procedure(s). Each intern was provided time to practice and received individualized feedback in a deliberate practice fashion.^5^

#### Day 2 – 9 hours of instruction

**Figure 3:**
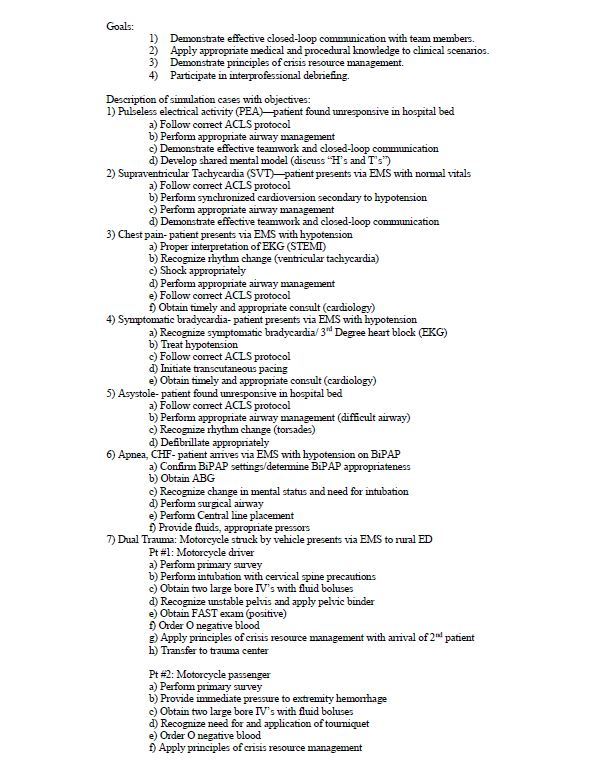
Goals and outline of capstone experience day 2.

At the beginning of day two, interns received an orientation to the high-fidelity simulators, resuscitation bay, ancillary equipment and staff available. They were informed that the simulation site functions as a learning environment and were encouraged to treat the simulation as realistically as possible. For the duration of the second day, six code scenarios and a final dual trauma were simulated, which required participants to apply several of the skills learned from the previous training (Figure 3).

In preparation for day two, materials were gathered including possible test results such as digital x-rays, electrocardiograms, ultrasound images and computed tomography scans to enhance the fidelity of the simulation. Each resuscitation bay was stocked with a crash cart, the appropriate trainers and equipment. This included an airway box, central line kit and trainer, thoracostomy tray with thoracostomy tubes, cricothyrotomy kit, tourniquet and pelvic binder. Most materials used during the code drills were recycled or expired to both save on costs and mirror the equipment residents would typically encounter in actual clinical environments. Each of the first six scenarios required four residents, two confederate nurses, a confederate paramedic and a high-fidelity adult simulator to mimic clinical conditions.^6^ The dual trauma scenario provided a final resuscitation opportunity in which the interns begin the scenario managing one critically injured geriatric trauma patient but are then confronted with another critical accident victim (Figure 3). After each scenario, the interns were debriefed using advanced debriefing strategies.^7^ The goals and objectives listed in Figure 3 were addressed in a formative nature during the debriefing process by several of the EM core faculty observing the simulations.

### Survey Questionnaire

Pre and post surveys were administered in order to gauge interns’ confidence levels and areas of weakness. In addition, we sought to identify specific areas in need of improvement within our boot camp curriculum. The pre survey was comprised of two sets of 12 questions aimed at outlining interns’ confidence levels and prior experience. The post survey consisted of 15 questions, 12 of which were repeat questions from the pre survey, along with three additional open-ended questions to identify strengths and weaknesses of the boot camp curriculum. This was a quality assurance project and did not meet the definition of human subject research and was therefore exempt from institutional review board review.

## RESULTS

This pilot study demonstrated that interns enter residency with an overall confidence score of 3.2 (1–5 scale) across all surveyed skills (Table 1). Interns reported the highest pre-survey confidence scores in suturing (4.3) and genitourinary exams (3.9). The lowest pre-survey confidence score was in thoracostomy (2.4). Following the capstone experience, overall confidence scores increased to 4.0. There was a significant improvement in confidence across eight procedures (Table 1). Confidence increased the most in defibrillation and thoracostomy (Table 1). Additionally, all interns reported post-survey confidence scores of at least 3.0 in all skills, representing an internal anchor of “moderately confident/need guidance at times to perform procedure.” Approximately 80% of interns had fewer than five simulation and five clinical experiences across all surveyed skills preceding the capstone event (Table 2).

## DISCUSSION

Our boot camp curriculum provided both a procedural component and a scenario-based component. The first day of the capstone experience emphasized fundamental procedural skills. The second day focused on the creation of a broad differential diagnosis, recognition of the correct procedure(s) to perform and use of stress mitigation strategies.^8^ Teamwork and leadership are critical components of diagnosing and providing care for patients with a wide range of high acuity illnesses. The diverse series of eight scenarios highlighted the value of each team member in terms of their knowledge and troubleshooting ability. Rotating the leadership position in each scenario provided interns with the opportunity to better envision role dynamics typical in clinical settings. Additionally, it emphasized the concept of closed-loop communication between interns, ultimately empowering the team to safely delegate responsibilities, stabilize patients and focus on different aspects of patient care.^9^ The end result of closed-loop communication, knowledge sharing and task delegation is to minimize the chance of complications due to miscommunication and system failure.^10^

Prior to the capstone experience, interns reported the highest confidence scores in suturing and genitourinary exams. This high level of confidence may be attributed to prior exposure during the course of their medical education, including practical experience on clinical rotations (Table 2). The low level of confidence in thoracostomy was likely due to the limited clinical exposure and opportunity to perform the procedure. Following the capstone experience, the significant rise in intern confidence scores suggests the effectiveness of our boot camp curriculum.

In addition to our quantitative results, interns’ written responses provided a framework through which the boot camp curriculum’s strengths and weaknesses could be evaluated. An appreciated aspect was the small group sizes. This allowed for greater access to faculty and more opportunities for individuals to practice their learned skills in a deliberate practice approach.^5^ Interns valued the hands on approach to the complex cases and the manner in which they were debriefed. They appreciated the constructive, team-based approach in addressing prospective clinical errors, highlighting the importance of having skilled debriefers. This concentrated, simulation-based curriculum aims to improve both procedural and leadership skills in a safe and effective learning environment.

## LIMITATIONS

This study has several limitations. First, the sample size was small and consisted of interns from one institution over one year. Second, we used a non-validated survey tool and confidence scores provide subjective data. While confidence scores may prospectively predict clinical performance, future studies should use more concrete assessment tools to objectively measure interns’ progress.^3^ Finally, many goals and objectives listed were not objectively evaluated with an instrument and were addressed during formative post-simulation debriefing sessions.

## CONCLUSION

At the conclusion of the EM boot camp curriculum, the interns had an improvement in self-reported confidence across all surveyed procedures and skills. The described EM boot camp curriculum was effective, feasible and provided a foundation to our trainees during their first month of residency. Our boot camp curriculum offers educators a framework from which they can implement their own training programs with coordinated effort, relatively inexpensive materials and dedicated faculty.

## Figures and Tables

**Table 1:** Pre- and post-survey confidence scores of interns participating in an emergency medicine boot camp.

Clinical skills	Pre survey confidence mean (standard deviation) 1–5 scale	Post survey confidence mean (standard deviation) 1–5 scale	p-value (significant <0.05)*
Genitourinary exam	3.9 (0.9)	4.4 (0.5)	0.11
Defibrillation	3.0 (1.3)	4.3 (0.5)	0.0059*
Intubation	3.7 (0.9)	4.0 (0.6)	0.35
Central lines	3.3 (1.0)	3.8 (0.6)	0.15
Slit lamp	2.7 (1.1)	3.8 (0.6)	0.0074*
Suturing	4.3 (0.7)	4.4 (0.5)	0.69
Thoracostomy	2.4 (1.2)	3.7 (0.7)	0.0046*
Lumbar puncture	3.0 (1.1)	3.8 (0.7)	0.045*
Arterial line	2.7 (1.2)	3.7 (0.8)	0.025*
Arterial blood gas	3.1 (1.2)	4.2 (0.9)	0.019*
Intraosseus	3.3 (1.1)	4.4 (0.7)	0.0079*
Code team leader	2.7 (1.2)	3.8 (0.6)	0.0095*
Overall	3.2 (0.56)	4.0 (0.29)	0.00012*

**Table 2:** Pre-survey frequency of clinical skills.

	Simulation			Clinically		
		
Clinical skills	<5 Times	5–10 Times	>10 Times	<5 Times	5–10 Times	>10 Times
Genitourinary exam	9/12 (0.75)	2/12 (0.17)	1/12 (0.083)	1/12 (0.083)	4/12 (0.33)	7/12 (0.58)
Defibrillation	6/12 (0.50)	4/12 (0.33)	2/12 (0.17)	8/12 (0.66)	3/12 (0.25)	1/12 (0.083)
Intubation	3/12 (0.25)	1/12 (0.083)	8/12 (0.67)	8/12 (0.66)	2/12 (0.17)	2/12 (0.17)
Central line	7/12 (0.58)	4/12 (0.33)	1/12 (0.083)	10/12 (0.83)	2/12 (0.17)	0/12 (0.00)
Slit lamp	12/12 (1.00)	0/12 (0.00)	0/12 (0.00)	10/12 (0.83)	0/12 (0.00)	2/12 (0.17)
Suturing	6/12 (0.50)	4/12 (0.33)	2/12 (0.17)	0/12 (0.00)	1/12 (0.083)	11/12 (0.92)
Thoracostomy	10/12 (0.83)	2/12 (0.17)	0/12 (0.00)	11/12 (0.92)	1/12 (0.083)	0/12 (0.00)
Lumbar puncture	8/12 (0.67)	4/12 (0.33)	0/12 (0.00)	12/12 (1.00)	0/12 (0.00)	0/12 (0.00)
Arterial line	11/12 (0.92)	1/12 (0.083)	0/12 (0.00)	9/12 (0.75)	3/12 (0.25)	0/12 (0.00)
Arterial blood gas	11/12 (0.92)	1/12 (0.083)	0/12 (0.00)	8/12 (0.66)	4/12 (0.33)	0/12 (0.00)
Intraosseus	9/12 (0.75)	2/12 (0.17)	1/12 (0.083)	12/12 (1.00)	0/12 (0.00)	0/12 (0.00)
Code team leader	8/12(0.66)	2/12 (0.17)	2/12 (0.17)	11/12 (0.92)	1/12 (0.083)	0/12 (0.00)
